# Network meta-analyses performed by contracting companies and commissioned by industry

**DOI:** 10.1186/s13643-016-0377-3

**Published:** 2016-11-25

**Authors:** Ewoud Schuit, John PA Ioannidis

**Affiliations:** 1 Stanford Prevention Research Center, Stanford University, 1265 Welch Road, Stanford, CA 94305 USA; 2 Meta-Research Innovation Center at Stanford (METRICS), Stanford University, 1070 Arastradero Road, Stanford, CA 94304 USA

**Keywords:** Industry, Contracting company, Network meta-analysis, Non-publication, Publication bias, Transparency, Protocol registration

## Abstract

**Background:**

Industry commissions contracting companies to perform network meta-analysis for health technology assessment (HTA) and reimbursement submissions. Our objective was to estimate the number of network meta-analyses performed by consulting companies contracted by industry, to assess whether they were published, and to explore reasons for non-publication.

**Methods:**

We searched MEDLINE for network meta-analyses of randomized trials. Papers were included if they had authors affiliated with any contracting company. All identified contracting companies as well as additional ones from the list of the exhibitors at the International Society for Pharmacoeconomics and Outcomes Research, an annual meeting that representatives from many contracting companies attend and exhibit at, were surveyed regarding conduct and publication of network meta-analyses.

**Results:**

In 162 of 822 (20%) network meta-analysis papers, authors were affiliated to 66 contracting companies. Another 36 contracting companies were identified by the exhibitors list. Three companies had no contact information and six merged with others, therefore 93 companies were contacted. Thirty seven out of ninety three (40%) companies responded, and 19 indicated that they had performed a total of 476 network meta-analyses, but only 102 (21%) papers were published.

Thirteen companies that disclosed to have conducted 174 network meta-analyses (45 published) provided reasons for non-publication. Of the 129 still unpublished meta-analyses, for 40 there were plans for future publication, for 37 the sponsor did not allow publication, for 16 the contracting companies did not plan to publish the meta-analysis, for another 23 plans were unclear, and the remaining 13 were used as HTA submission.

The protocol of the network meta-analysis was publically available from 11/162 (6.8%) network meta-analyses published by authors affiliated with contracting companies.

**Conclusions:**

There is a prolific sector of professional contracting companies that perform network meta-analyses. Industry commissions many network meta-analyses, but most are not registered before or published after analyses in the scientific literature. Mechanisms to improve publication rates of network meta-analysis commissioned by industry are warranted.

**Electronic supplementary material:**

The online version of this article (doi:10.1186/s13643-016-0377-3) contains supplementary material, which is available to authorized users.

## Background

For many clinical conditions, multiple treatment options are assessed in randomized trials. Using network meta-analysis, it is possible to assess the comparative effectiveness of multiple interventions using both direct and indirect evidence [1–3]. Network meta-analyses are particularly useful for clinical guideline development and policy, and to quantify relative treatment effects (and eventually ranking multiple treatments options for efficacy and/or safety) and the uncertainty around these effects in order to inform cost-effectiveness analyses (CEA) and therefore healthcare resource allocation decisions. Development of methods for and the use of network meta-analysis has been strongly driven by regulatory requirements of health technology assessment (HTA) and reimbursement submissions in general and CEA in particular, as HTA requires the comparison of a novel drug against a broad range of marketed comparators to ensure that the therapies with the most favourable benefit/risk profile reach patients and that limited healthcare resources are appropriately invested. Additionally, these requirements initiated the development of an industry of people conducting these studies in private firms, so-called contracting consulting companies. These contracting companies are commissioned by biotech and pharmaceutical industry to conduct network meta-analysis to facilitate HTA and reimbursement submissions to agencies such as the National Institute for Health and Care Excellence (NICE) or Canadian Agency for Drugs and Technologies in Health (CADTH).

Network meta-analysis has grown rapidly, especially over the last few years [4]. An increasing number of contracting consulting companies undertake meta-analyses and several of them perform also network meta-analyses. The exact extent of this phenomenon has not been systematically studied. It would be interesting to understand who performs these analyses and how they get disseminated or not in the literature. We anticipate that many more meta-analyses, and network meta-analyses in particular, have been conducted than those published. In commissioned analyses, non-publication may occur for several reasons, including but not limited to unfavorable results for the manufacturer, unwillingness to share with the public (and thus also with competitors) private information and/or information that they consider important to give them insights and strategic advantages, low priority for publication for meta-analysis topics that might have already been covered in other published papers, or simply no strong incentives for the manufacturer (i.e., in the form of regulation) or contracting company to publish the results.

The goal of this study was to determine the amount of network meta-analyses performed by consulting companies contracted by industry. At a second stage, we aimed also to explore whether the results of these meta-analyses were published and, if not, why they remain unpublished.

## Methods

### Strategy

Network meta-analyses can be performed by contracting companies commissioned by the industry to perform this work. Two searches were performed to identify these contracting companies. Afterwards, these companies were sent surveys with questions related to the number of performed network meta-analyses, number published, and reasons for non-publication.

### Identification of contracting companies: sources and searches

First, MEDLINE was searched for network meta-analyses from inception until 6 May 2015. We used the following search strategy: “(network [tiab] AND (meta analys*[tiab])) OR indirect comparison*[tiab] OR indirect treatment comparison*[tiab] OR multiple treatment comparison*[tiab] OR mixed treatment comparison*[tiab]”. No language restrictions were used. Papers were considered potentially eligible if they described a network meta-analysis of randomized clinical trials. We did not exclude papers that apart from randomized clinical trials also included quasi-randomized clinical trials or papers in which they combined data from observational studies and randomized clinical trials. Papers describing non-intervention studies, letters to the editor, editorials, protocols, reviews, and papers describing methodology were excluded. Papers that used a network meta-analysis to assess cost-effectiveness but not clinical effectiveness or safety were also excluded. The potentially eligible papers were scored by one author (ES) based on the affiliations of the authors for being conducted by academia (university or hospital), industry (biotech or pharmaceutical company), contracted consulting company, or a combination.

Second, the list of the exhibitors at the 20th Annual International Meeting (May 2015) of the International Society for Pharmacoeconomics and Outcomes Research (ISPOR) was searched for contracting companies [5]. The ISPOR is an international multidisciplinary professional membership society, that aims to advance the policy, science, and practice of pharmacoeconomics (health economics) and outcomes research (the scientific discipline that evaluates the effect of health care interventions on patient well-being including clinical, economic, and patient-centered outcomes). As such, many professionals from academia, industry, and consulting companies attend ISPOR meetings, and many (consulting) companies have exhibitions to promote their companies and to recruit personnel and attract new customers. For those companies of the ISPOR list for which it was unclear whether they perform network meta-analysis, inclusion was based on the information on the company’s website (identified through Google by ES).

No further selection of contracting companies occurred. The company was considered for further study if a person affiliated to a contracting company co-authored a published network meta-analysis or if their company website indicated that they did perform network meta-analysis.

### Data collection via surveys

Identified contracting companies from both searches were contacted by e-mail or through a contact form on the company’s website if an e-mail address was not available. Companies were surveyed and asked: (1) has your company ever undertaken any network meta-analyses, and if so, (2) when did your company first perform a network meta-analysis, (3) how many network meta-analyses have been completed by your company to date, where a network meta-analysis referred to a commissioned network meta-analysis (which may in fact include more than one network meta-analysis, e.g. for different outcomes, or subgroups), (4) how many of these completed network meta-analyses have been published in the literature, (5) if they could list references of network meta-analyses that were published? If we had identified network meta-analyses through our literature search we sent the company the reference (s) of the paper (s) and asked whether they could list any additional papers. Each company was informed that we planned to publish the results and was allowed to have their company’s name be de-identified in the publication, if they wished so. Companies were contacted three times with e-mails sent 2 weeks apart. For companies that were now part of another company, we contacted the mother company to provide information about the former company. When representatives responded that they had no time to provide an exact number of conducted and published network meta-analyses they were allowed to give an estimate. Papers that were quoted as “published” according to the companies but were in fact conference abstracts (*N* = 23 meta-analyses), no network meta-analysis (*N* = 4), cost-effectiveness analysis (*N* = 2), or a methodological paper (*N* = 2) were not considered as published network meta-analyses.

After collection of the data on this survey, we sent out an additional survey to those companies that had responded that they do perform network meta-analysis. Here, we asked for specific reasons of network meta-analyses not being published: (1) paper was submitted but has been rejected, no plan to resubmit, (2) paper was submitted, but has been rejected, still trying to publish, (3) paper was submitted and is under peer review (first submission), (4) paper will be submitted in the future, (5) no plan to publish the network meta-analysis, (6) not allowed by the pharma sponsor to publish the network meta-analysis, or (7) other reason (specify). Again, companies were contacted three times with e-mails sent 2 weeks apart and conference abstracts were not considered published papers and were considered not published with reason “unclear”.

Papers that we initially did not assign to a contracting company because none of the authors on the paper had the company’s affiliation (*N* = 9), but that which were listed by a company were included in the total number of published papers by that company. Additionally, papers that we did assign to a company based on author affiliations in the publication, but that were not listed by the company itself in its reply (*N* = 29) were also included in the total number of published papers by that company.

For network meta-analyses with a contracting company affiliation, we investigated whether a protocol was publically available. The protocol could either be referenced to in the paper or could be identified through PROSPERO [6] or the website of the journal of publication, reimbursement agency, or research institute. We also looked at protocol availability of network meta-analyses published 1 year after the launch of PROSPERO (Feb 2011) [7] to take into account the lower awareness of the value of registry of systematic reviews and meta-analyses before their launch.

### Data analysis

Data analysis was descriptive. Quantitative data are presented with median and interquartile ranges and categorical data with number and percentages.

## Results

### Identification of contracting companies

**Fig. 1:**
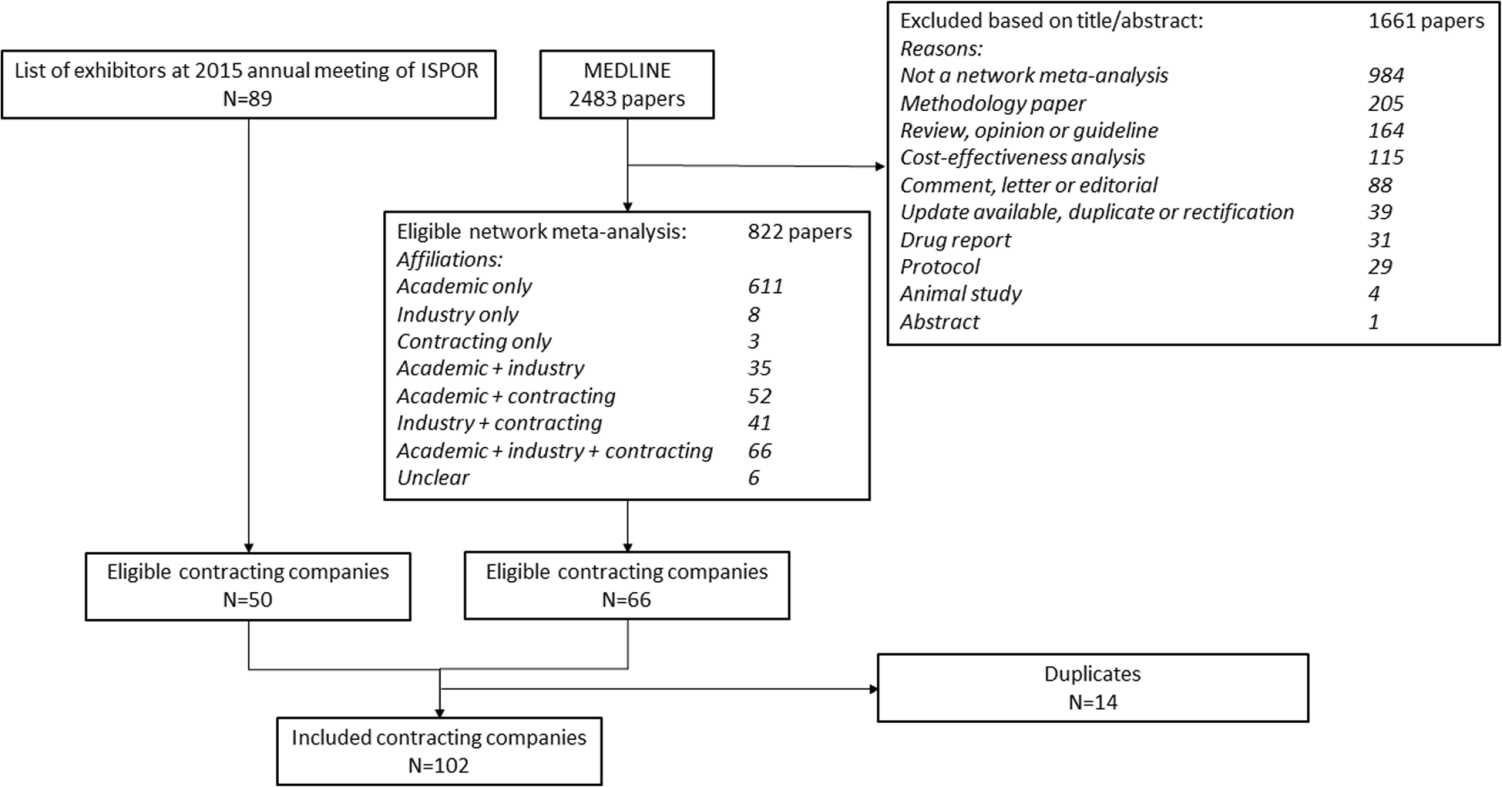
Flow diagram of identification of network meta-analyses and of contracting companies

The search, study selection, and identification of eligible contracting companies are presented in Fig. 1.

Through our database search, we identified 2483 papers of which 822 presented a network meta-analysis based on title and abstract screening. Authors were affiliated to a contracting company in 162 (20%). Contracting company affiliations were rarely the sole affiliations of the authors of these network meta-analyses; typically, the papers had also authors affiliated to industry or academia or both. In 35 papers (22%), at least one of the authors with a contracting company affiliation also had an academic affiliation, and in one paper, two authors had both a contracting company and industry affiliation. From this literature search, 66 contracting companies were identified.

The list of exhibitors at the 2015 Annual Meeting of ISPOR contained 89 listings, and of those, 50 (36 not already identified by the literature search) were identified as eligible for inclusion in our study.

A total of 102 contracting companies were thus identified (see Additional file 1). For three companies, no contact information was available, including one company that was dissolved. Another six companies were incorporated into other companies, leaving 93 eligible contracting companies for inclusion in the e-mail survey.

### Survey

**Fig. 2:**
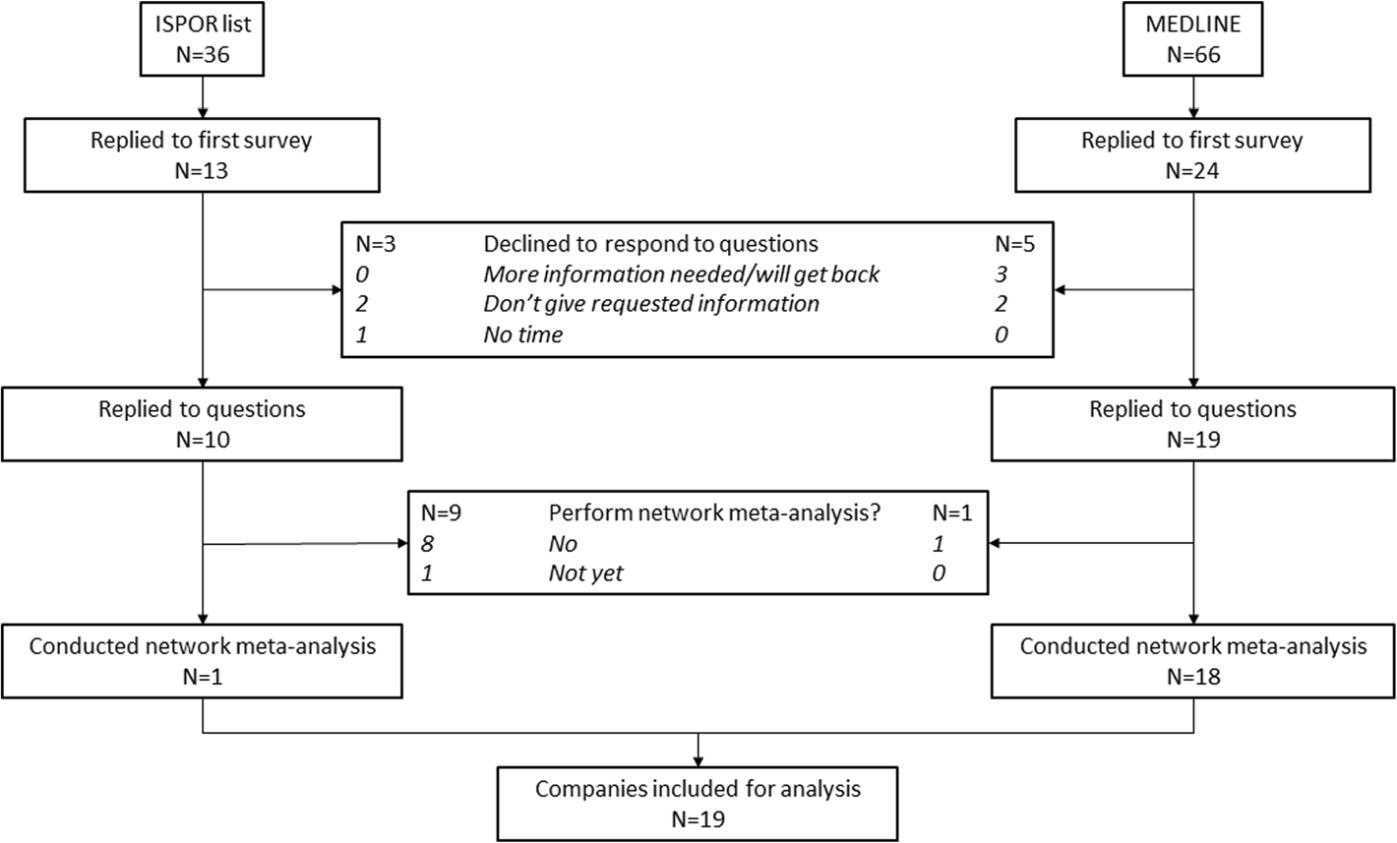
Flow diagram for contracting companies based on the replies to the first survey

Thirty-seven out of 93 contacted contracting companies (40%) responded to our first survey (Fig. 2). Twenty-nine responded fully, and 19 indicated they had performed network meta-analysis. The 19 companies that responded to our survey were involved in 89 of the 162 network meta-analysis papers that we had identified upfront. These 19 companies were actually involved in 102 published network meta-analysis papers (the 89 we identified, plus another 13 provided to us by the companies—two papers not indexed in PubMed, two not identified by our search, and nine where the publication did not have a contracting company affiliation, but which nevertheless were claimed by the company). Four of the 102 papers had authors from two contracting companies each.

### First survey

The replies of the 19 companies to our first survey are presented in Table 1. The experience of the companies with these analyses differed largely as indicated by the starting date of first network meta-analysis (range: June 2000—Sept 2013) and the number of network meta-analyses that they claimed to have performed (range: 1—100). Together the 19 companies made claims to the performance of a total of 476 network meta-analyses. The number of independent network meta-analyses may be slightly smaller, since some of those may have involved more than one contracting company. Four companies gave an estimate of the number of conducted network meta-analyses rather than an exact number. The average number of conducted network meta-analyses per year per company was 6.6 for the total sample, but varied widely between companies (range 0.16–36).

**Table 1:** Results of the 19 contracting companies that indicated that they perform network meta-analysis

	Company	Start date of first NMA	NMAs completed	NMAs published	NMA conducted/year	NMA published/year	Ratio published/conducted
1	Mapi Group (incl. Health Technology Analysts and Optum)	Nov-05	88	33	9.5	3.6	0.38
2	Abacus International/Decision Research Group	Jun-07	54	10	7.0	1.3	0.19
3	Redwood Outcomes (now part of Precision Health Economics)	Sept-13	51	5	36	3.5	0.10
4	Evidera/UBC^c^	Nov-08	50	3	8.0	0.48	0.06
5	Kleijnen Systematic Reviews Ltd^c^	Jan-08	20	14	2.8	2.0	0.70
6	Amaris	Jan-12	15	1	4.9	0.32	0.07
7	Symmetron Ltd	Nov-08	13	2	2.1	0.32	0.15
8	RTI Health Solutions	Jun-00	12	13	0.82	0.89	1.08
9	Medignition Inc.	Aug-08	6	6	0.92	0.92	1.00
10	Augmentium Pharma Consulting	Jan-05	5	4	0.50	0.40	0.80
11	AHEAD	Jun-10	2	2	0.43	0.43	1.00
12	Xintera Consulting	Jan-09	2	2	0.33	0.33	1.00
13	CEMKA-EVAL	May-12	2	0	0.73	0.0	0.0
14	McMDC Ltd	Sep-09	2	1	0.37	0.18	0.50
15	Pharmacoeconomics and Outcomes Research Iberia	Jan-10	2	1	0.39	0.20	0.50
16	BeSyRe Bekkering Systematic Reviews	May-10	1	1	0.21	0.21	1.00
17	David Hoaglin (consulting statistician)	Jan-09	1	1	0.16	0.16	1.00
18	Company 1^c^	NP	NP	NP	NP	NP	0.04
19	Company 2^c^	NP	NP	NP^a^	NP	NP	0.08
	Total	–	476^b^	106^b^	6.6	1.5	0.22

Companies are sorted based on the number of network meta-analyses (NMA) conducted

*NP* not presented due to confidentiality

^a^Since no list of references was available from Company 2, we used the number of papers identified by our literature search

^b^Meta-analyses conducted or published by more than one contracting company are double-counted, but these are uncommon. The sum of 106 for published network meta-analyses corresponds to 102 papers, because four meta-analyses were co-authored by two contracting companies. The exact level of redundancy for conducted meta-analyses is not correct, but is likely to be similarly low

^c^These companies gave an approximation for the number of conducted network meta-analyses. For calculations the approximation was considered as the actual number of network meta-analyses

Companies provided evidence that they had published between 0 and 33 network meta-analyses each for a sum of 106 (102 independent papers, four papers were co-authored by two companies). The ratio of 106 publication contributions versus 476 claims of network meta-analyses conducted is 0.22 and the proportion of published vs. conducted network meta-analyses varied widely between companies (range 0–1.08), with one company for which we found more published than claimed conducted network meta-analyses (13 vs. 12; ratio published/conducted = 1.08). The overall number of network meta-analyses published per year was 1.5 and was found to vary also widely between companies (range 0–3.6).

### Second survey

Sixteen out of 19 companies replied to our second survey. Of these, two companies did not keep specific information about publication status, and one company indicated they had no time to complete the survey. Together with the three companies that did not respond, these six were responsible for 302 (63%) out of the total 476 conducted network meta-analyses.

Based on the replies by the 13 companies that did provide reasons for non-publication, 45 out of 174 (26%) conducted network meta-analyses had already been published. Up to another 40 network meta-analyses maximum could perhaps be published in the near future, as the authors indicated they were or would be pursuing plans for publication: two meta-analyses had already been submitted but were not published yet but the company indicated they were still trying to publish, 10 were currently under review, and the company indicated plans for future publication for another 28 meta-analyses. Two meta-analyses had been submitted, but were rejected and the company indicated no future plans to pursue publication. The sponsor was claimed to be responsible for not allowing publication of 37/129 non-published network meta-analyses. For another 14 meta-analyses, there was no plan to publish, but the reason was not stated. A further 13 meta-analyses had been used for health technology assessment (HTA) submissions to reimbursement agencies with unclear plans for further publication in the scientific literature. Finally, for 23 meta-analyses it was unclear whether there was a plan to publish them or not. When we added the 302 network meta-analyses conducted by the six companies that did not respond to our second survey, the reason for non-publication was unclear in (23 + 241)/476 (55%) of all meta-analyses. The proportion of published network meta-analyses in companies that provided reasons for non-publication was not significantly different from those that did not respond (45/174 (26%) vs. 61/302 (20%), *p* = 0.15 by chi-square test).

### Protocol availability

Of the 162 network meta-analyses with one or more authors affiliated with a contracting company, 10 (6.2%) referred to a protocol, 33 (20%) mentioned a protocol was drafted but did not provide a reference, and 119 (73%) did not refer to or mention a protocol in their paper. When limited to network meta-analyses published after Feb 2012 (1 year after the launch of PROSPERO), these numbers changed to 10 (9.1%), 23 (21%), and 77 (70%), respectively, out of 110 network meta-analyses. Overall, we managed to identify and access the protocol of 11 (6.8%) network meta-analyses. Three were available through PROSPERO (all *stage of review* “ongoing”), five through the National Institute for Health Research (NIHR) of which one was also registered in PROSPERO, two through the Agency for Healthcare Research and Quality (AHRQ), and one each through the Cochrane Collaboration, and the National Institute for Health and Care Excellence (NICE). One network meta-analysis used a company website of the York Health Economics Consortium (YHEC) to refer to the protocol, but the website was no longer available. All published network meta-analyses with a publically available protocol had at least one co-author with a sole academic affiliation. For one network meta-analysis, the protocol was published after the date of the last search listed in the paper (July 13, 2011 (AHRQ website protocol) vs. Jan 2011 (AHRQ website research review)). Publication of the final paper did occur after the publication date of the protocol (December 5, 2011).

## Discussion

Overall, our evaluation found a total of 102 companies that provide consultancy in evidence synthesis. Nineteen of these contracting companies replied to our first survey disclosing that they had conducted many hundreds of network meta-analyses, but the results were published for only a small minority. Among the contracting companies that replied to our second survey, there was an intent to publish about half of the meta-analyses in the peer-reviewed literature and some others have been used for HTA submissions. Unwillingness of the industry sponsor to allow publication was the most common specified reason for lack of a plan for publication. Registration of meta-analysis protocols was found to be poor.

To our knowledge, this is the first effort to investigate the number of network meta-analyses performed by contracting companies commissioned by the industry. There are, however, some limitations to our study. First, the response rate of the addressees was 40%. This is probably acceptable given the involvement of rather sensitive information [8]. Probably many non-responders did not actually perform network meta-analysis. Second, since 4 companies out of the 37 that responded were unable to provide information due to confidentiality agreements with industry, it is possible that some other companies did not respond at all for this same reason. Nevertheless, the 19 companies that did perform network meta-analyses were responsible for the majority of all published network meta-analyses affiliated with a contracting company, therefore probably we did capture most of the main stakeholders operating in this field. The 74 published network meta-analyses for which no company replied to our first survey were affiliated to 48 different contracting companies, indicating that the non-responders had published few network meta-analyses each and were either less involved in this type of work and/or had more prominent non-publication rates than the responders. Third, the accuracy of the information provided by survey responders cannot be fully verified. Juxtaposition against verifiable information, e.g., number of published papers, suggested mostly good concordance, but we cannot verify information on unpublished work and the reasons for its lack of publication.

So far, evidence for non-publication in evidence-based medicine has focused primarily on randomized trials rather than meta-analyses. Low publication rates have been found for randomized clinical trials in many fields, [9–13] and specifically in industry-sponsored trials [9, 10, 12]. Industry-sponsored trials that did get published have been shown to more often find a treatment effect in benefit of the sponsor’s treatment [14, 15]. Additionally, examples exist of intentional withholding of trial reports from publication due to results not in favor of the sponsor’s drug [16]. This may also apply to network meta-analyses. Sponsors may know the outcomes where their drugs rank high and ask for a network meta-analysis on these outcomes. Furthermore, contracting companies have no incentive to spend resources on analyzing outcomes other than the ones requested. There is also a high risk of presenting results in a way that favors the sponsor’s drug. Further challenges may arise when publically unavailable data is included in meta-analyses initiated by industry.

In our study, veto from the industry was the most common specific stated reason for not having a plan for publication of network meta-analyses. Moreover, a substantial number of meta-analyses were left with unclear reasons for non-publication even among the responders, so the proportion of these evidence syntheses that were not published because of the unwillingness of the industry sponsor may be higher. Especially, considering that the six contracting companies responsible for two-thirds of all conducted network meta-analyses did not provide reasons for non-publication. It is unknown whether the decision for non-publication was made before or after seeing the results and thus whether non-publication reflects the presence of unfavorable results for the manufacturers, unwillingness to share with the public (and thus also with competitors) private information with strategic advantages, or low priority for publishing meta-analyses on topics already covered in other published papers [17]. In general, we think it is safe to state that publication of any type of analysis done for industry cannot be separated from the overall commercial strategy and is not usually done with the aim of disseminating generalizable knowledge.

Moreover, contracting companies that are commissioned to perform network meta-analyses do not necessarily have strong incentives to publish the results, even without any veto from the industry sponsor. Contractors may not have the “publish or perish” pressure that is typical of academic investigators. Manuscript preparation and publication also require resources that a profit-oriented business may not want to spend. Lack of peer-reviewed publication has been seen also in other areas of biomedical investigation where entrepreneurs are involved for profit and has been termed stealth research [18]. In fact, we identified very few published network meta-analyses where contracting companies were the only type of affiliation. The majority of those published had also academic affiliations which would offer a traditional motivation for pursuing publication.

Some network meta-analyses that were not published in the peer-reviewed journal literature were part of an HTA submission to regulatory agencies like NICE or the Canadian Agency for Drugs and Technologies in Health (CADTH). Nevertheless, these submissions generally only present selected results, while specific details are sent to the agency separately, thereby making it very difficult to assess the rigor and validity of the network meta-analysis and its results. Similarly, some pharmaceutical companies may publish their results on websites or at conferences, but again, judging the quality and inclusiveness of the analyses is difficult.

Non-transparency is further compounded by lack of protocol registration. The percentage of protocol registration was low, especially when you consider that close to 400 network meta-analysis protocols and more than 12,000 systematic review and/or meta-analysis protocols have been registered on PROSPERO. Prospective registration of systematic reviews with written protocols [6, 7] is increasingly endorsed [19]. The Cochrane Comparing Multiple Interventions Methods Group (CMIMG) has developed protocol templates and has made these protocols available on their website [20]. Protocol registration will not avoid the need for unanticipated deviations from the protocol, but would make deviations more visible and open to public judgment [21, 22].

We did not try to estimate the volume of traditional systematic reviews and pairwise meta-analyses conducted and/or published by contractors, but this is also likely to be impressive. Our survey clearly shows there is a huge market for evidence synthesis, even for the most sophisticated type of synthesis method. A large segment of this work happens outside of peer-reviewed publications, with results only known to contractors and their sponsors.

Non-publication of network meta-analysis results seems particularly problematic as network meta-analysis is an evidence synthesis technique that allows informing health policy and guidelines. Regardless of the exact reasons, non-publication results in a largely non-transparent corpus of evidence synthesis work that would otherwise have been of potential value to patients, clinicians and guideline developers, and even the industry itself. Reimbursement agencies may require industry to preregister a protocol, e.g., in PROSPERO, before accepting their HTA submissions. Another way to improve publication rates may come from regulatory bodies that could require certain information about the HTA submission to be made publically available, similar to the transparent reporting for systematic reviews and meta-analyses (PRISMA) extension for network meta-analyses (PRISMA-NMA standards [23]).

## Conclusions

There is a prolific sector of professional contracting companies that perform network meta-analyses. Industry commissions many network meta-analyses, but most are not registered before or published after analyses in the scientific literature. Mechanisms to improve publication rates of network meta-analysis commissioned by industry are warranted.

## Additional file

Additional file 1: List of eligible contracting companies. The additional file lists eligible contracting companies, whether they were identified via MEDLINE or the ISPOR exhibitors list, and whether they responded to our first survey. (DOC 111 kb)
