# Supplementary material for: Network meta-analyses performed by contracting companies and commissioned by industry
Source: Syst Rev. 2016 Nov 25;5:198. doi: 10.1186/s13643-016-0377-3 (PMC5123429; doi:10.1186/s13643-016-0377-3)
Supplement: Additional file 1: — List of eligible contracting companies. The additional file lists eligible contracting companies, whether they were identified via MEDLINE or the ISPOR exhibitors list, and whether they responded to our first survey. (DOC 111 kb) [file 13643_2016_377_MOESM1_ESM.doc]

Additional file 1. List of eligible contracting companies.

| **Company name** | **Source**  **0 = ISPOR list**  **1 = PubMed** | **Response** |
| --- | --- | --- |
| ***Included*** |  |  |
| Abacus International - Decision Resources Group (soon DRG) | 1 | Yes |
| AHEAD - Agency for Health Economic Assessment and Dissemination | 1 | Yes |
| Amaris | 1 | Yes |
| AMICULUM | 0 | Yes |
| Amygdala Ltd | 1 |  |
| Analysis Group | 1 |  |
| Ars Accessus Medica | 1 |  |
| Augmentium Pharma Consulting | 1 | Yes |
| BCN Health | 0 |  |
| Berry Consultants | 1 |  |
| BeSyRe Bekkering Systematic Reviews | 1 | Yes |
| BioPoint Inc. | 0 |  |
| Boston Health Economics | 0 |  |
| Boston Strategic Partners | 0 |  |
| BresMed Health Solutions | 1 |  |
| Business & Decision | 1 | Yes |
| Catalyst Health Economics Consultants | 0 |  |
| CEMKA-EVAL | 1 | Yes |
| Context Matters | 0 | Yes |
| Continuum Clinical | 0 |  |
| ConvergeHEALTH by Deloitte | 0 |  |
| Corinne LeReun, consulting statistician | 1 |  |
| Cornerstone Research Group Inc. | 0 | Yes |
| Covance | 1 |  |
| Creativ-Ceutical SARL | 1 |  |
| CRECON Medical Assessment Inc. | 0 |  |
| David Hoaglin, consulting statistician | 1 | Yes |
| Deloitte | 1 |  |
| DOCS International | 1 |  |
| Double Helix Consulting | 1 |  |
| ECRI Institute | 0 | Yes |
| Elysia Group | 1 |  |
| eMAX Health | 0 |  |
| Enhance Reviews | 1 |  |
| Envision Pharma Group | 0 | Yes |
|  |  |  |
| **Company name** | **Source**  **0 = ISPOR list**  **1 = PubMed** | **Response** |
| ***Included*** |  |  |
| EPI-Q, Inc. | 0 | Yes |
| EpiX Analytics | 1 | Yes |
| ESiOR Oy | 1 |  |
| Evaluation Network for Health Economics (REES) | 1 |  |
| EVIDENCIAS | 1 |  |
| Evidera  /UBC | 1 | Yes |
| Exponent | 0 | Yes |
| Fondazione Charta | 0 | Yes |
| GalbraithWight | 0 |  |
| GfK | 0 |  |
| Ghement Statistical Consulting | 1 | Yes |
| i3 Innovus | 1 |  |
| Icera Consulting | 1 |  |
| ICON Commercialisation & Outcomes | 1 | Yes |
| IHS | 0 | Yes |
| IMS Health | 1 |  |
| Inovalon, Inc. | 0 |  |
| Institute of empirical health economics | 0 |  |
| Institute for Health Economics | 0 |  |
| Kantar Health | 0 |  |
| Keyrus Biopharma | 1 |  |
| Kleijnen Systematic Reviews Ltd | 1 | Yes |
| LASER Analytica | 0 |  |
| Laurent Eckert, independent consultant | 1 |  |
| Mapi | 1 | Yes |
| Market Access Solutions (MKTXS) | 0 |  |
| Matrix Knowledge Group (now Optimity Matrix) | 1 |  |
| McMDC Ltd | 1 | Yes |
| Medical Decision Modeling | 1 | Yes |
| Medignition Inc. | 1 | Yes |
| Mediprobe CRO phase II-IV trials | 1 | Yes |
| Novosys Health | 0 |  |
| PAREXEL | 1 |  |
| Pharmacoeconomics & Outcomes Research Iberia | 1 | Yes |
| Pharmerit International | 1 |  |
| PharmIdeas Research and Consulting Inc. | 1 |  |
| Pharsight Consulting Services (owned by Certara) | 1 |  |
| **Company name** | **Source**  **0 = ISPOR list**  **1 = PubMed** | **Response** |
| ***Included*** |  |  |
| PHT Corporation | 0 |  |
| Policy Analysis Inc. | 0 |  |
| Precision for Value | 0 |  |
| Precision Health Economics (owned by Precision for Value) | 0 | Yes |
| PRMA Consulting | 0 | Yes |
| qPharmetra | 1 | Yes |
| Quantics | 1 |  |
| Quintiles | 1 |  |
| Redwood Outcomes (now part of Precision Health Economics) | 1 | Yes |
| RTI Health Solutions | 1 | Yes |
| SHE-consulting | 1 |  |
| Spectrum Research Inc. | 1 |  |
| STATinMED Research | 0 |  |
| Symmetron Ltd | 1 | Yes |
| Tolley Health Economics Consultancy Ltd | 1 |  |
| Trinity Partners, LLC | 0 | Yes |
| Truven Health Analytics | 0 |  |
| Vegter Health Economic Research | 1 |  |
| Xcenda | 1 |  |
| Xintera Consulting | 1 | Yes |
| York health consortium | 1 | Yes |
| ZRx Outcomes Research | 0 |  |
| *Companies not contacted/taken over* |  | Company included? |
| Fourth Hurdle Consulting | 1 | No, cannot find contact details, dissolved |
| Fullarton Consultancy | 1 | No, cannot find contact details |
| Strategen Ltd | 1 | No, cannot find contact details |
| MTAG | 1 | Yes, as part of IMS Health |
| Analytica International | 1 | Yes, as part of LASER |
| Health Technology Analysts | 1 | Yes, as part of MAPI |
| Optum | 0 | Yes, as part of MAPI |
| Oxford Outcomes Ltd. | 1 | Yes, as part of ICON |
| Heron Health | 1 | Yes, as part of Parexel |
